# Trends in Emergent Groin Hernia Repair—An Analysis From the Herniamed Registry

**DOI:** 10.3389/fsurg.2021.655755

**Published:** 2021-03-30

**Authors:** Ferdinand Köckerling, Till Heine, Daniela Adolf, Konstaninos Zarras, Dirk Weyhe, Bernhard Lammers, Franz Mayer, Wolfgang Reinpold, Dietmar Jacob

**Affiliations:** ^1^Department of Surgery and Center for Minimally Invasive Surgery, Academic Teaching Hospital of Charité Medical School, Vivantes Hospital, Berlin, Germany; ^2^COPV – Hernia Center, Berlin, Germany; ^3^StatConsult GmbH, Magdeburg, Germany; ^4^Department of Visceral, Minimally Invasive and Oncologic Surgery, Academic Teaching Hospital of University of Düsseldorf, Marien Hospital, Düsseldorf, Germany; ^5^Pius Hospital, Department of General and Visceral Surgery, University Hospital of Visceral Surgery, Oldenburg, Germany; ^6^Department of Surgery I, Section Coloproctology and Hernia Surgery, Lukas Hospital, Neuss, Germany; ^7^Department of Surgery, Paracelsus Medical Private University Salzburg, Salzburg, Germany; ^8^Department of Surgery, Wilhelmsburger Hospital Groß Sand, Academic Teaching Hospital of University Hamburg, Hamburg, Germany

**Keywords:** groin hernia, bowel resection, mortality, emergency, perioperative complications

## Abstract

**Introduction:** While the proportion of emergency groin hernia repairs in developed countries is 2.5–7.7%, the percentage in developing countries can be as high as 76.9%. The mortality rate for emergency groin hernia repair in developed countries is 1.7–7.0% and can rise to 6–25% if bowel resection is needed. In this present analysis of data from the Herniamed Registry, patients with emergency admission and operation within 24 h are analyzed.

**Methods:** Between 2010 and 2019 a total of 13,028 patients with emergency admission and groin hernia repairs within 24 h were enrolled in the Herniamed Registry. The outcome results were assigned to the year of repair and summarized as curves. The total patient collective is broken down into the subgroups with pre-operative manual reduction (taxis) of the hernia content, operative reduction of the hernia content without bowel resection and with bowel resection. The explorative Fisher's exact test was used for statistical assessment of significant differences with Bonferroni adjustment for multiple testing.

**Results:** The proportion of emergency admissions with groin hernia repair within 24 h was 2.7%. The percentage of women across the years was consistently 33%. The part of femoral hernias was 16%. The proportion of patients with pre-operative reduction (taxis) remained unchanged at around 21% and the share needing bowel resection was around 10%. The proportion of TAPP repairs rose from 21.9% in 2013 to 38.0% in 2019 (*p* < 0.001). Between the three groups with pre-operative taxis, without bowel resection and with bowel resection, highly significant differences were identified between the patients with regard to the rates of post-operative complications (4% vs. 6.5% vs. 22.7%; *p* < 0.0001), complication-related reoperations (1.9% vs. 3.8% vs. 17.7%; *p* < 0.0001), and mortality rate (0.3% vs. 0.9% vs. 7.5%; *p* < 0.001). In addition to emergency groin hernia repair subgroups female gender and age ≥66 years are unfavorable influencing factors for perioperative outcomes.

**Conclusion:** For patients with emergency groin hernia repair the need for surgical reduction or bowel resection, female gender and age ≥66 years have a highly significantly unfavorable influence on the perioperative outcomes.

## Introduction

Every year around 20 million hernia operations are carried out across the world (1). The vast majority of groin hernia operations are performed electively and are considered to be low-risk procedures (2). Groin hernias can give rise to an emergency if patients develop an incarceration or strangulation of the hernia sac contents (3). Incarceration happens if it is no longer possible to reduce the hernia sac contents because the hernia defect is too narrow or due to adhesions (3). In the case of strangulation the blood flow to the organs within the hernia sac (e.g., omentum, bowel) is squeezed off (3). If the hernia sac contents cannot be safely reduced in such a situation (taxis) (4, 5), or there is a risk of recurrent strangulation, emergency repair is needed immediately (2, 3). While the proportion of emergency groin hernia repairs in developed countries is 2.5–7.7%, the proportion in developing countries can be as high as 76.9% (6, 7).

For femoral hernias the proportion of emergency repairs is markedly higher in developed countries, too, for both men at around 10.5% and, in particular, women at around 25.4% (8). Whereas, following elective groin hernia repair the mortality rate in developed countries is very low at 0.1% (2), after emergency operation it is between 1.7 and 7.0% (9, 10). If bowel resection is needed in emergency groin hernia repair, the mortality rates rise to 6–25% (11–14).

As treatment for an acutely incarcerated or strangulated groin hernia the HerniaSurge guidelines recommend a tailored approach, since there is no evidence supporting an optimal surgical approach (15).

In this present analysis the patient cases documented in the Herniamed Registry with emergency admission to the hospital and operation within 24 h were analyzed under various aspects. Separate analysis was performed for the years 2010–2019 to identify trends.

## Methods

Herniamed is an internet-based hernia registry in which 737 hospitals and surgeons in Germany, Austria, and Switzerland voluntarily entered data on all their routine hernia operations (16–18). All patients signed a consent form agreeing to their data being processed by Herniamed (16–18). As part of the information provided to patients (regarding participation in the Herniamed Registry), they are told that the treating hospital or the treating surgeon would like to be kept informed about any problem occurring after groin repair (16–18). If problems occur after the operation, the patient should contact the treating hospital or surgeon. Appropriate diagnostic measures should then be taken if needed (16–18).

All perioperative complications are documented for up to 30 days after the operation (16–18). Here, the intraoperative, post-operative general, and post-operative surgical complications as well as the complication-related reoperations are recorded separately (16–18).

After 1, 5, and 10 years patients and their general practitioner are sent a questionnaire by the treating hospital or surgeon (16–18). This questionnaire enquires once again about any post-operative complications (16–18). The questionnaire also asks about any pain at rest, pain on exertion, or pain requiring treatment (16–18). Patients are also asked whether they have already experienced hernia recurrence or observed any protrusion in the hernia repair region (16–18). If the patient or general practitioner reports a relevant finding, the patient may be requested by the treating hospital or surgeon to attend for further diagnostic examination.

All patients admitted to hospital in the years 2010–2019 as emergency cases with groin hernia and operated on within 24 h were included in the present analysis. On retrospective analysis of the prospectively collected data, different subgroups were distinguished:

1. Patients with emergency admission and operation within 24 h who, following reduction (taxis) of the hernia sac contents, no longer showed evidence of incarceration or strangulation but who were immediately operated on to prevent recurrent incarceration/strangulation.
2. Patients with emergency admission and operation within 24 h who still experienced incarceration or strangulation at the time of repair and for whom operative reduction of the incarceration or strangulation was possible without bowel resection.
3. Patients with emergency admission and operation within 24 h for whom, despite operative reduction of the incarcerated or strangulated hernia sac contents, bowel resection was needed.

Treatment and outcomes were analyzed separately for the subgroups for the years 2009–2019.

The surgical techniques transabdominal preperitoneal patch plasty (TAPP), totally extraperitoneal patch plasty (TEP), Lichtenstein and Shouldice techniques recommended in the HerniaSurge Guidelines (15) are presented separately, showing their rates of use in the various subgroups. All other techniques were summarized under the definition “other techniques.”

For statistical calculation of significant differences the explorative Fisher's exact test was used with an alpha = 5%. For *post-hoc* tests of single categories, a Bonferroni adjustment for multiple testing was implemented (18). Since the number of cases entered into the Herniamed Registry in the years 2010–2012 was still relatively small, major fluctuations in outcomes were observed for these years. Therefore, trend analysis was based on the more stable data collected over the years 2013–2019 (18). That applied for the perioperative outcome. Since follow-up was still not available for 2019 at the time of analysis, the pain and recurrence rates for the years 2013–2018 were compared (18).

## Results

**Figure 1:**
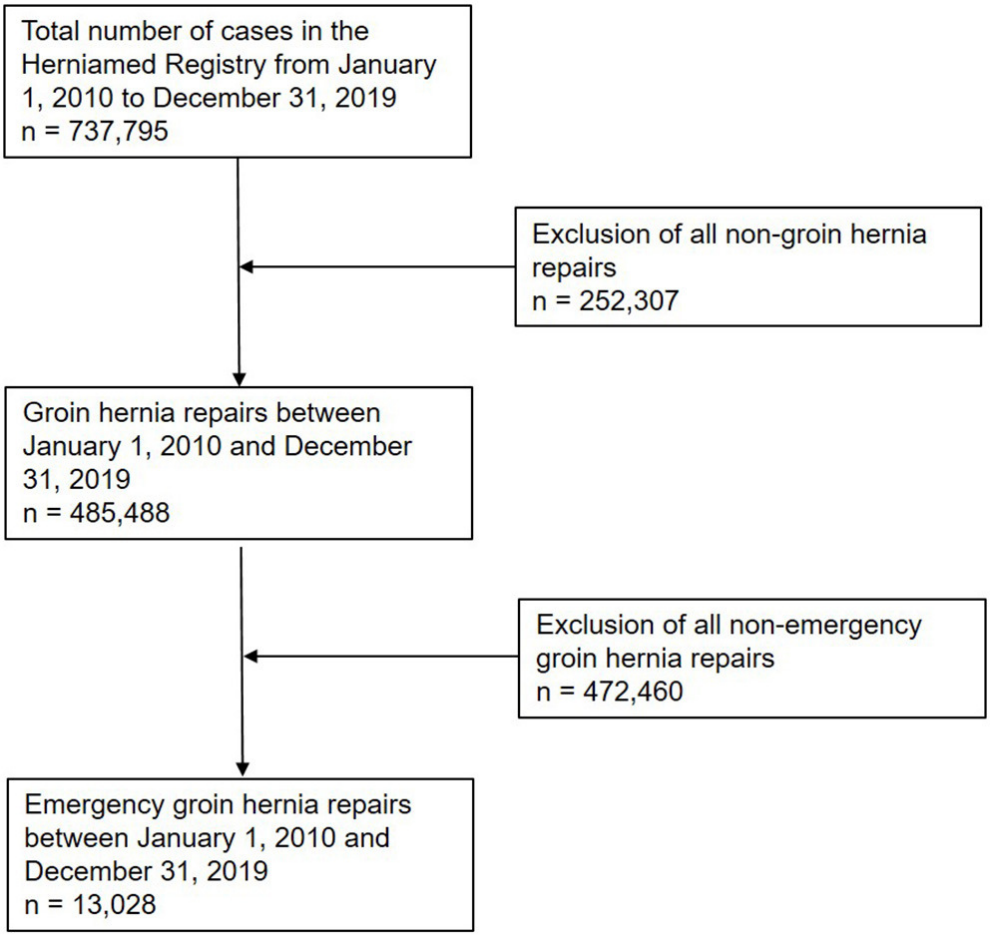
Flowchart of patient inclusion.

Between January 1, 2010 and December 31, 2019, a total of 13,028 cases with emergency admission and operation within 24 h were documented in the Herniamed Registry. That corresponds to a proportion of 2.7% of all the groin hernia repairs recorded in Hermiamed (Figure 1). The rise in the number of annual cases was due to an increase in the number of hospitals and surgeons participating in the Herniamed Registry (*n* = 176 in 2010, *n* = 1,554 in 2015, *n* = 2,201 in 2019).

**Figure 2:**
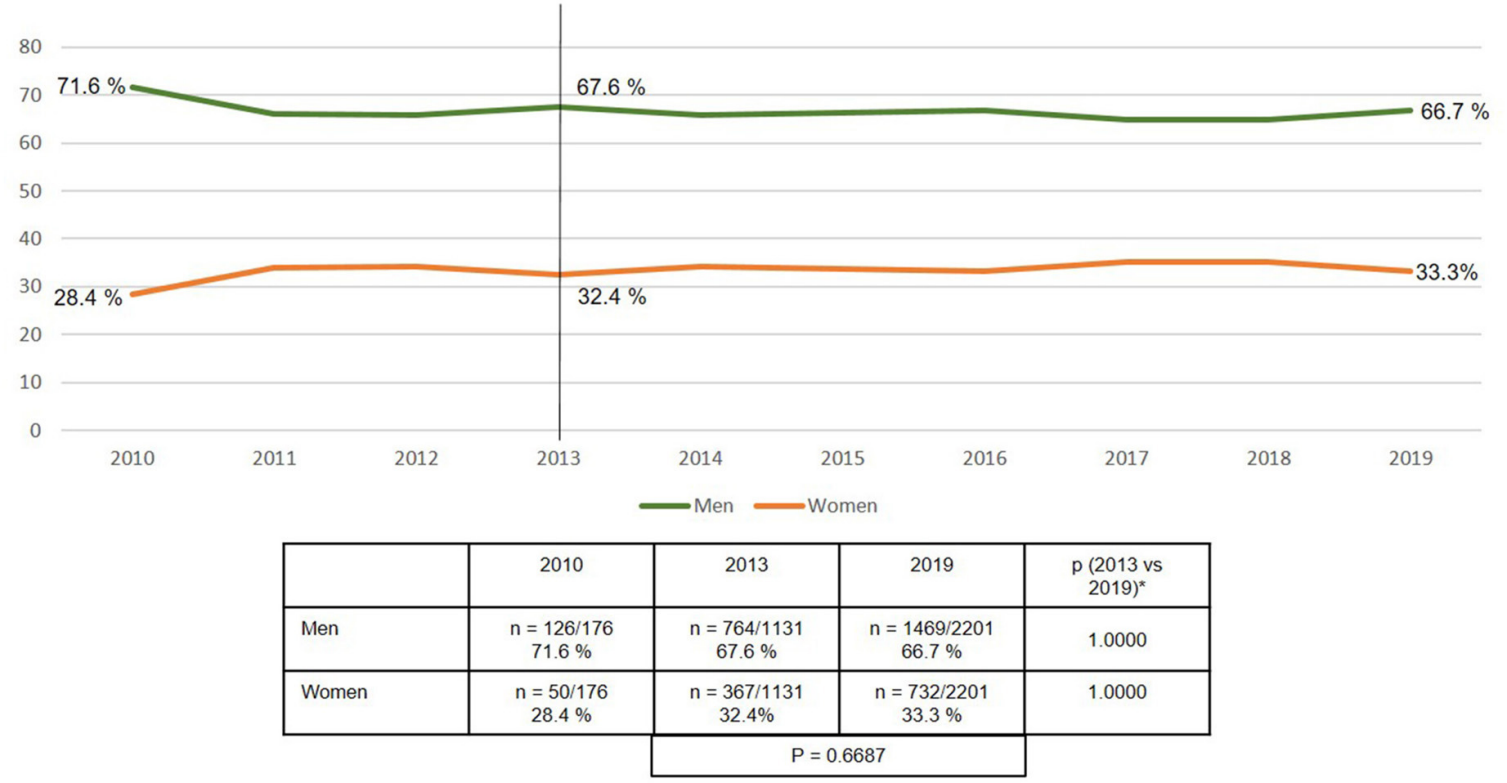
Gender of emergency groin hernia repair patients (*n* = 13,028) (2010–2019). *Bonferroni-adjusted (factor 2) for multiple testing.

The proportion of women in the total collective of emergency admissions and operation within 24 h remained stable over the years at 33% (Figure 2).

**Figure 3:**
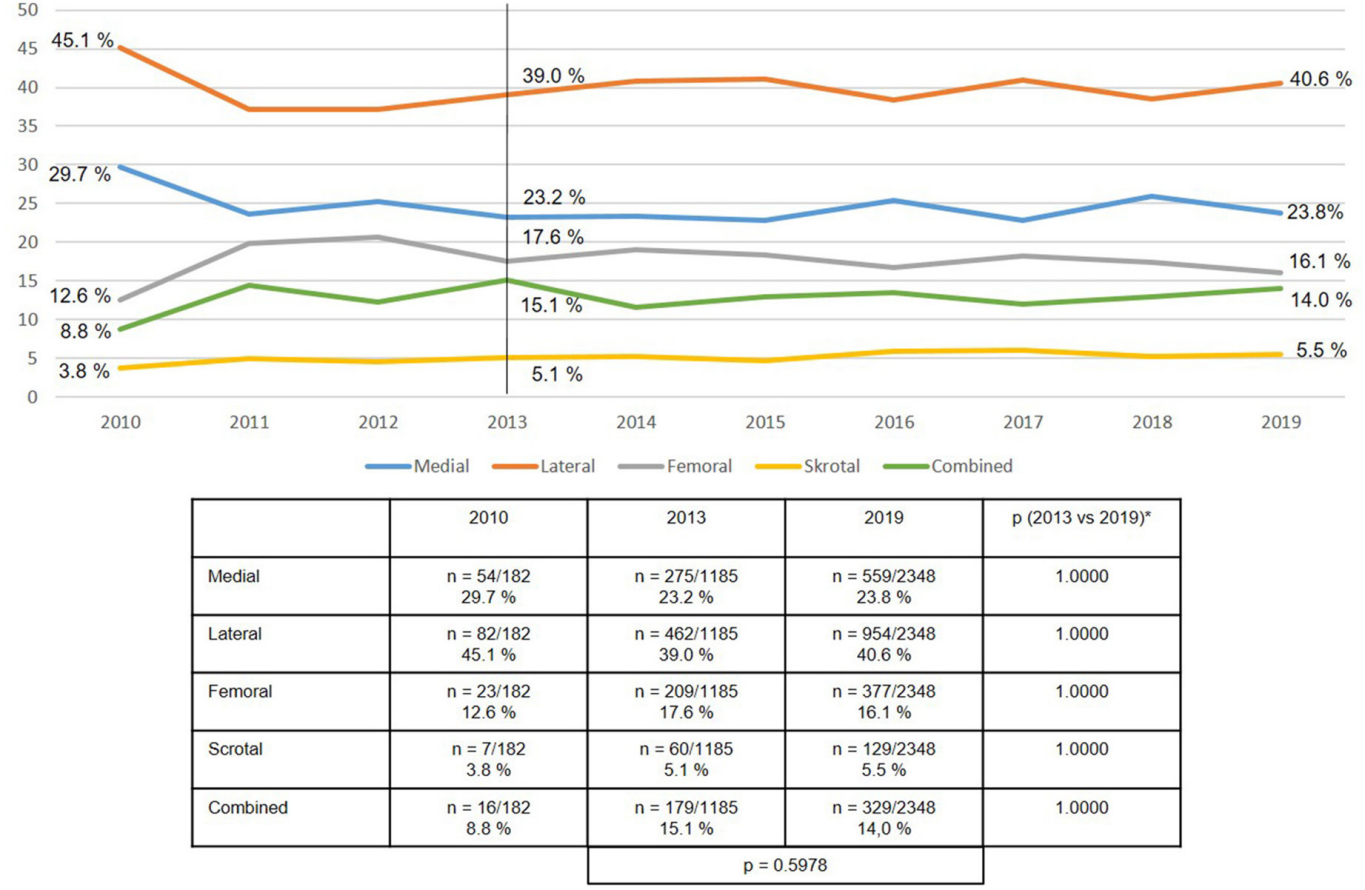
Defect localization in emergency groin hernia repairs using EHS classification (*n* = 13,717 procedures) (2010–2019). *Bonferroni-adjusted (factor 5) for multiple testing.

Similarly, the defect localization remained relatively stable over the years, with around 41% lateral, 24% medial, 16% femoral, 14% combined, and 5% scrotal defect localizations (Figure 3).

**Figure 4:**
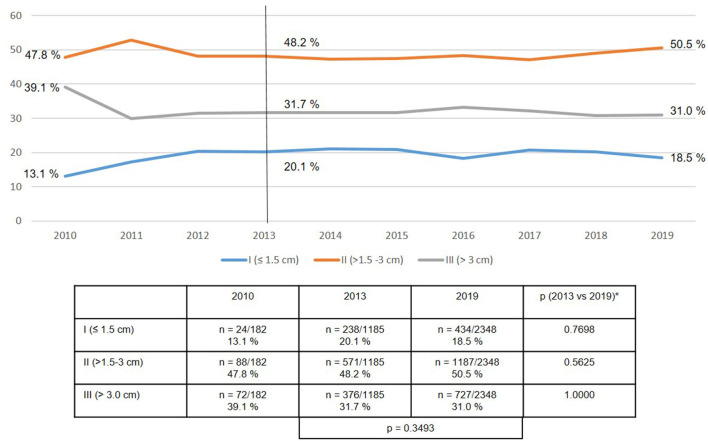
Defect size in emergency groin hernia repairs using EHS classification (*n* = 13,717 procedures) (2010–2019). *Bonferroni-adjusted (factor 3) for multiple testing.

In terms of the defect sizes, the most common continued to be defect sizes >1.5–3 cm at 50%, followed by defects >3 cm at 31%, and small defects ≤1.5 cm at 19% (Figure 4).

As regards all the emergency admissions with operation within 24 h, the most commonly used technique was the Lichtenstein operation at 40.1%, followed by TAPP at 29.7%, TEP at 9.2% and the Shouldice operation at 3.8%. Surgical techniques not recommended in the guidelines were relatively common at 17.2% including Bassini 2.1%, Plug 1.3%, Gilbert 0.3%, TIPP 1.0%, reduction of hernia sac 3.4%, and various other procedures 9.1 % (15).

**Figure 5:**
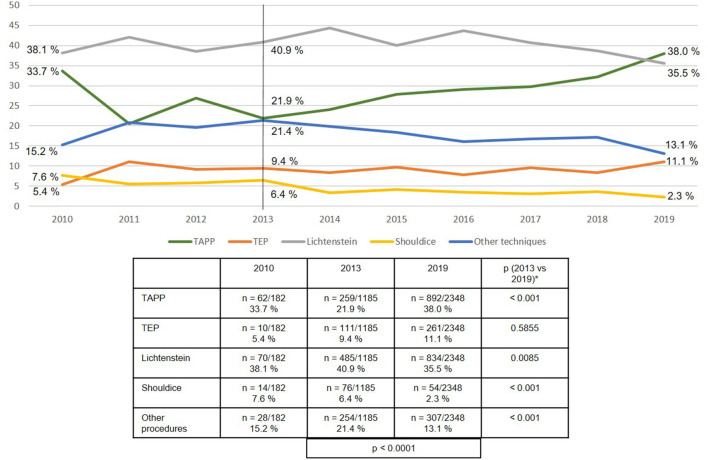
Emergency groin hernia repairs (*n* = 13,028 patients/*n* = 13,717 procedures) in different techniques (2010–2019). *Bonferroni-adjusted (factor 5) for multiple testing.

Looking at developments over the years 2010 to 2019, TAPP was used increasingly more often (21.9% in 2013 vs. 38.0% in 2019; *p* < 0.001), whereas the Lichtenstein technique, Shouldice operation and “other techniques” were used less frequently often (Figure 5).

**Figure 6:**
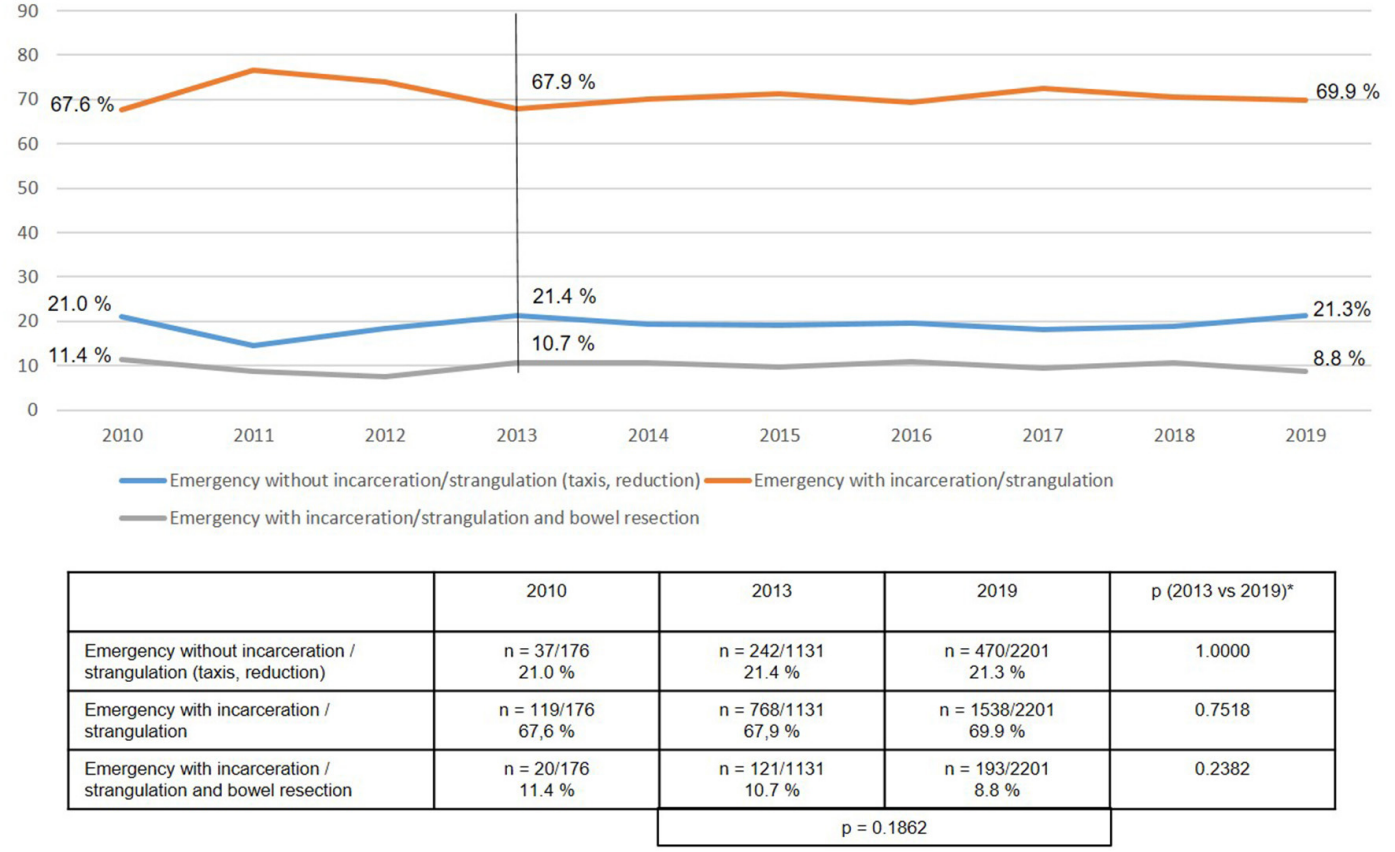
Subgroups of patients with emergency groin hernia repairs (*n* = 13,028) (2010–2019). *Bonferroni-adjusted (factor 3) for multiple testing.

Likewise, retrospective assignment of patients with emergency admission and operation within 24 h to the subgroups did not significantly change between 2013 and 2019 (Figure 6). Patients who at the time of emergency operation 24 h after admission no longer showed evidence of incarceration or strangulation following reduction (taxis) of the hernia sac contents, but who were immediately operated on to prevent recurrent incarceration/strangulation, consistently accounted for around 21% of all emergency procedures. In around 70% of cases it was possible to surgically reduce the incarceration or strangulation without the need for bowel resection. Only in around 9% of cases was bowel resection performed.

**Figure 7:**
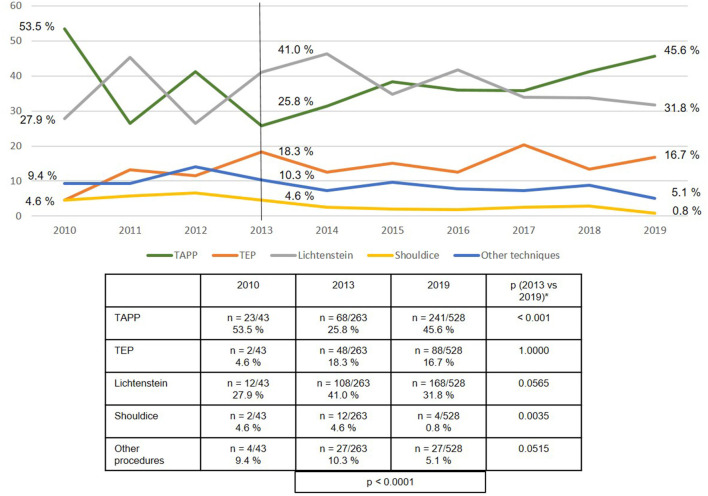
Emergency groin hernia repairs (*n* = 2,789) for patients without incarceration/strangulation (taxis, reduction) with different techniques (2010–2019). *Bonferroni-adjusted (factor 5) for multiple testing.

**Figure 8:**
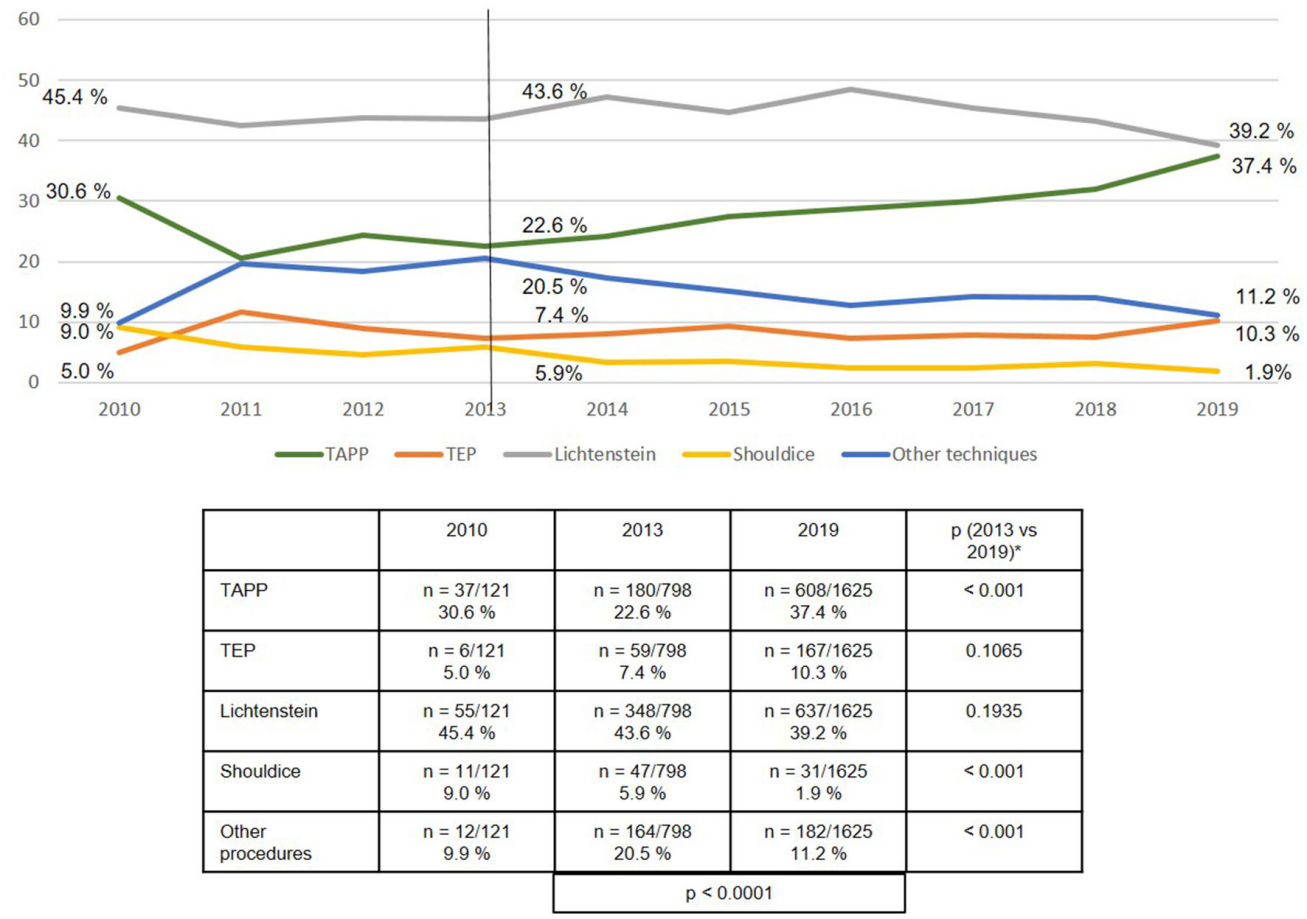
Emergency groin hernia repairs (*n* = 9,608) for patients with incarceration/strangulation without bowel resection in different techniques (2010–2019). *Bonferroni-adjusted (factor 5) for multiple testing.

The use of the various surgical techniques differed between the three subgroups. In the case of the patients with emergency operation after reduction/taxis of the hernia sac contents, the proportion of TAPP repairs rose highly significantly from 25.8 to 45.6%, whereas the proportion of Lichtenstein, Shouldice, and “other techniques” declined (Figure 7). Similarly, among the emergency operations without bowel resection the proportion of TAPP repairs increased significantly, whereas the proportion of Shouldice and “other techniques” decreased (Figure 8).

**Figure 9:**
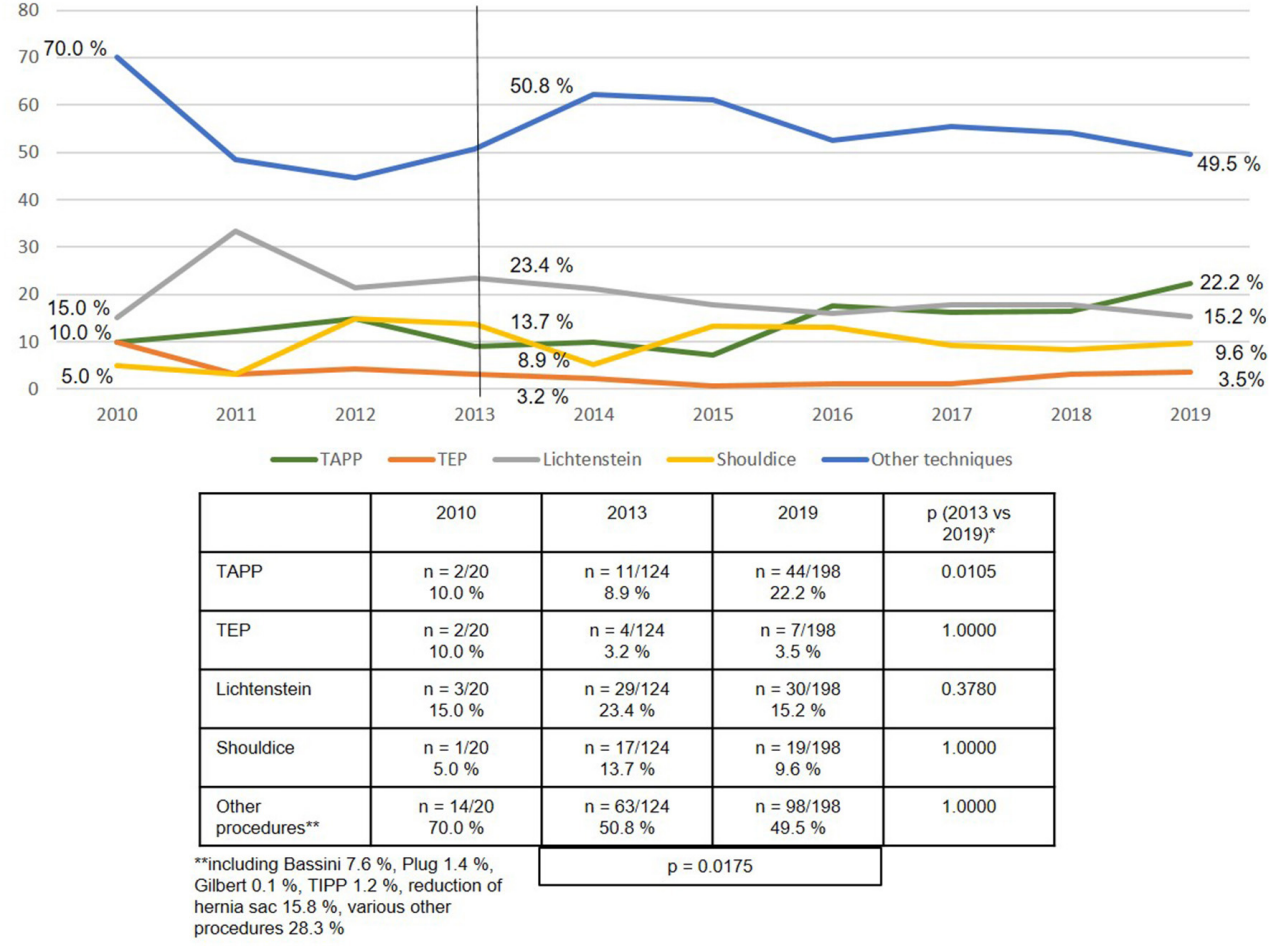
Emergency groin hernia repairs (*n* = 1,320) for patients with incarceration/strangulation and bowel resection with different techniques (2010–2019). *Bonferroni-adjusted (factor 5) for multiple testing.

While the proportion of TAPP repairs increased also for the emergency operations with bowel resection, “other techniques” predominated here at around 50% (Figure 9).

**Figure 10:**
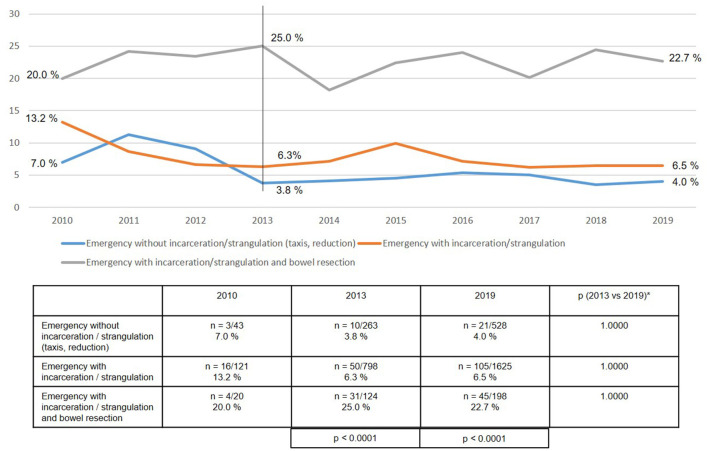
Post-operative surgical complications of emergency groin hernia repairs in different subgroups (*n* = 13,717) (2010–2019). *Bonferroni-adjusted (factor 3) for multiple testing.

**Figure 11:**
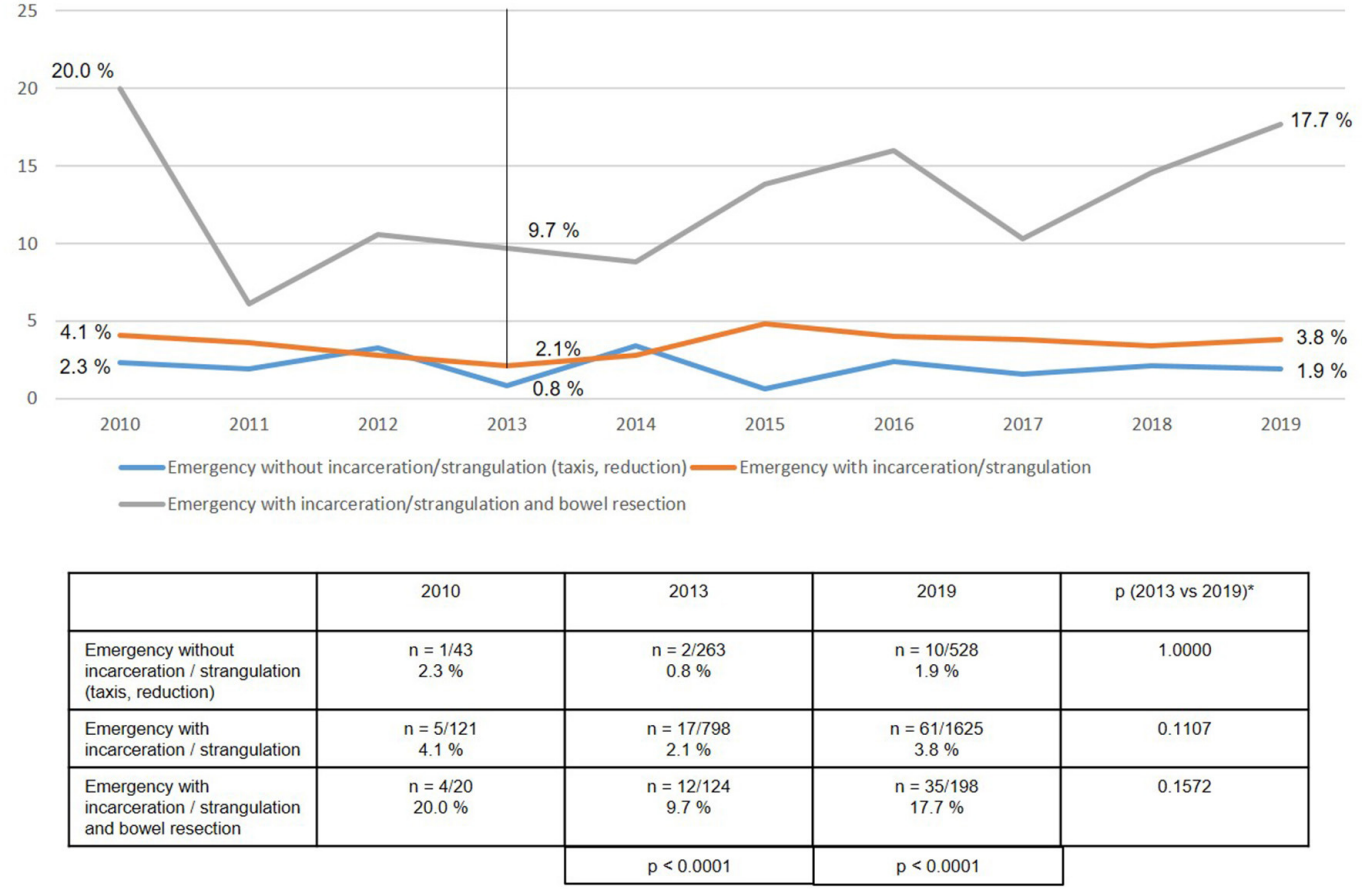
Complication-related reoperation rate of emergency groin hernia repairs (*n* = 13,717) in different subgroups (2010–2019). *Bonferroni-adjusted (factor 3) for multiple testing.

**Figure 12:**
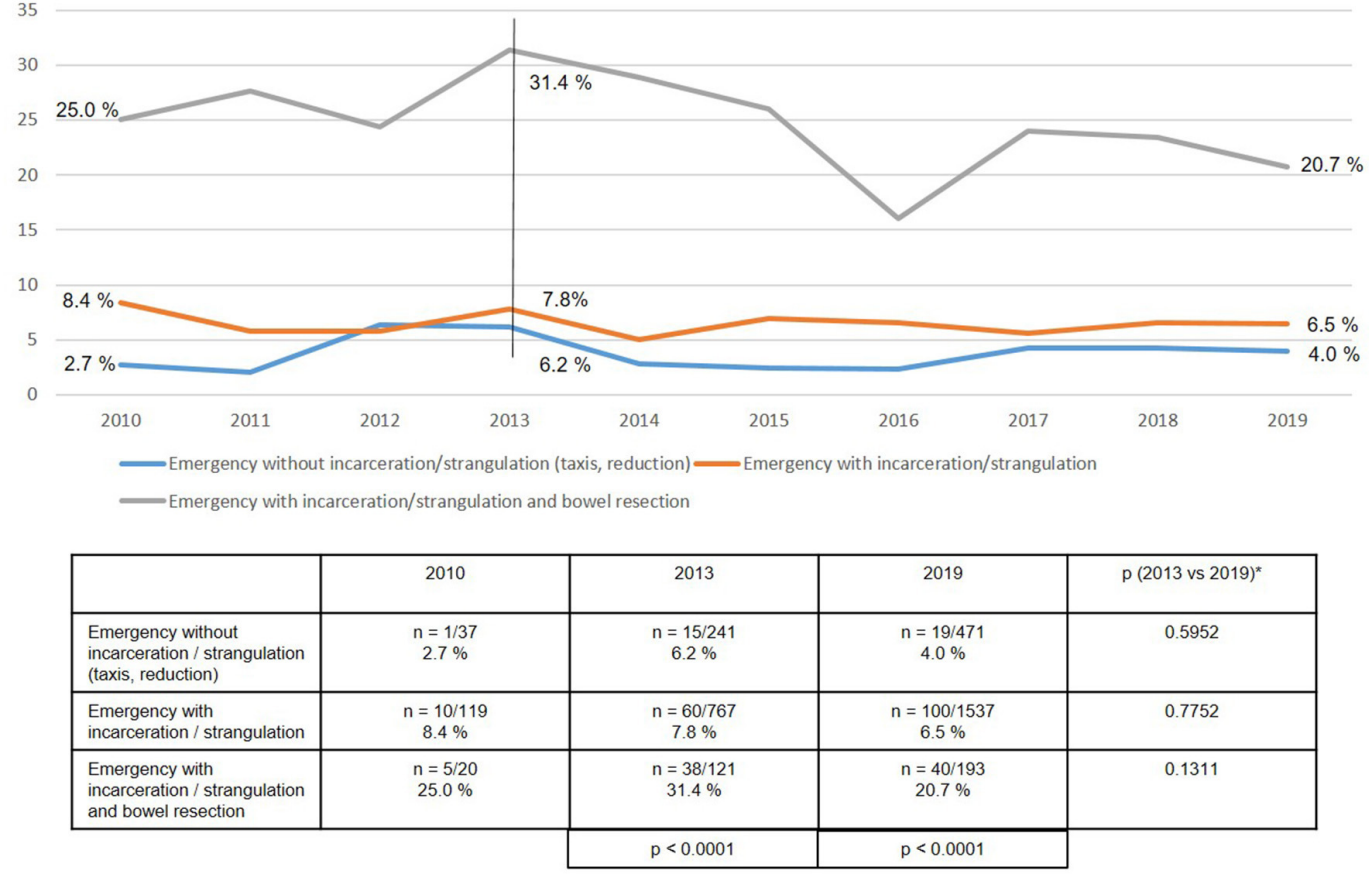
General complications in emergency groin hernia patients (*n* = 13,028) in different subgroups (2010–2019). *Bonferroni-adjusted (factor 3) for multiple testing.

**Figure 13:**
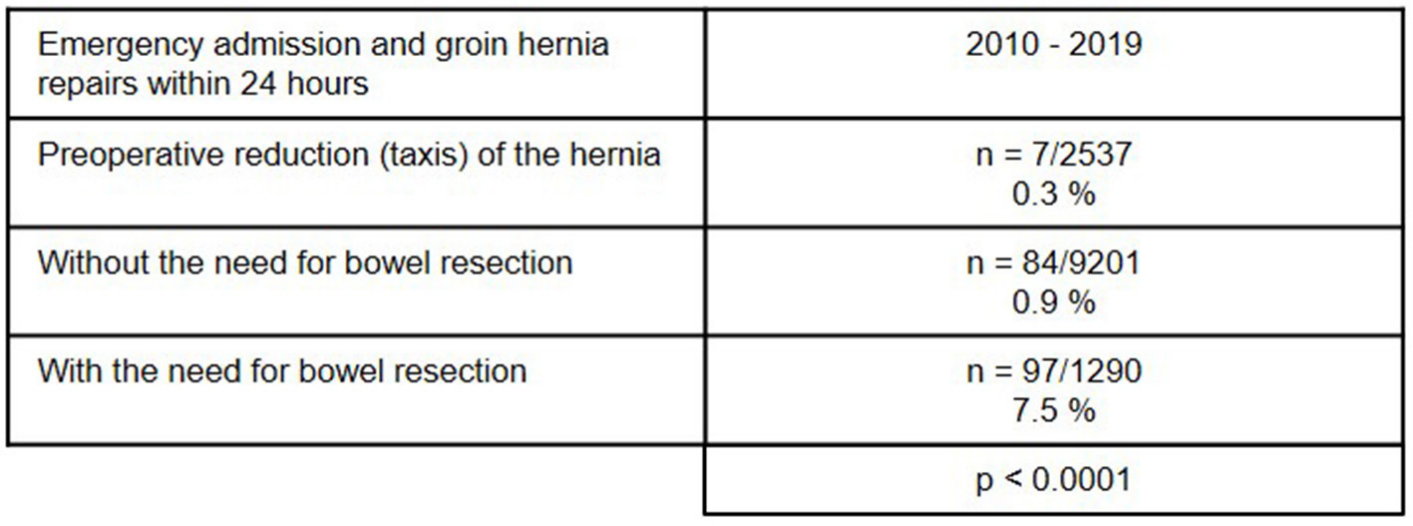
Mortality rate of emergency admissions and groin hernia repairs within 24 h (*n* = 13,028) in different subgroups (2010–2019) (total *n* = 188/13,028; 1.44%).

As regards the perioperative outcome, highly significant differences were identified between the subgroups (Figures 10–13). The post-operative surgical complication rates, complication-related reoperation rates, post-operative general complication rates, and the mortality rates were significantly lower in patients following (pre-operative) reduction/taxis of incarcerated or strangulated groin hernia than in patients following emergency operation of incarcerated or strangulated groin hernias without bowel resection. The highest perioperative complication rates were identified for emergency operation of an incarcerated or strangulated groin hernia needing bowel resection. Here, the mortality rate was 7.5% (Figure 13).

Additionally to the subgroups of emergency groin hernia repair, female vs. male gender has a highly significantly unfavorable impact on post-operative general complications (10.2% vs. 6.7%; *p* < 0.001), post-operative surgical complications (9.5% vs. 7.4%; *p* < 0.001), complication-related reoperations (5.2% vs. 3.7%; *p* < 0.001), and mortality (2.0% vs. 1.2%; *p* = 0.0004). Also patients age ≥66 years vs. ≤65 years show significantly higher rates of post-operative general complications (9.9% vs. 2.6%; *p* < 0.001), post-operative surgical complications (9.5% vs. 4.9%; *p* < 0.001), complication-related reoperations (5.0% vs. 2.4%; *p* < 0.001), and mortality (1.9% vs. 0.05%; *p* < 0.001).

**Figure 14:**
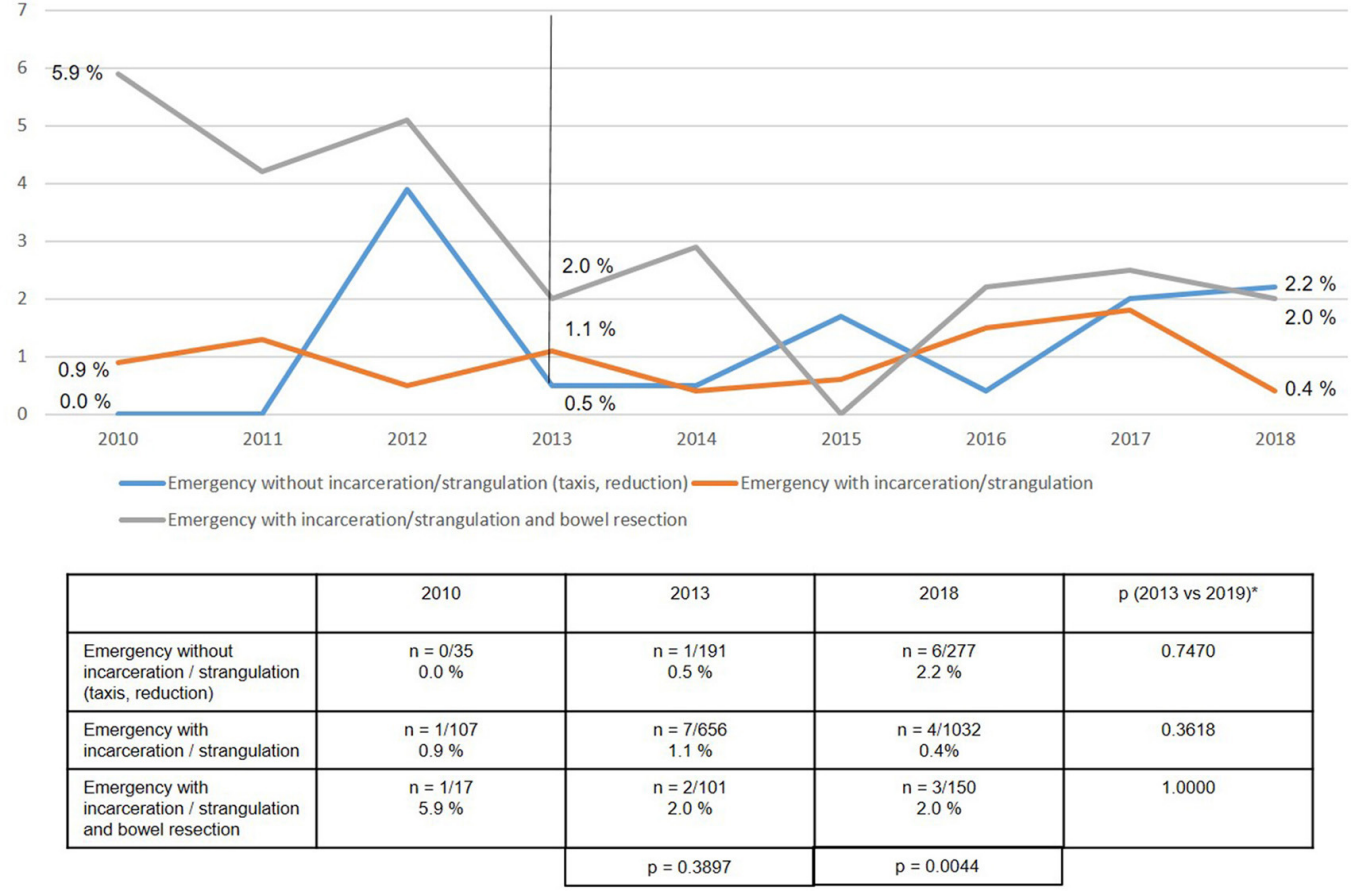
Recurrence rate at 1-year follow-up of emergency groin hernia patients (*n* = 8,303 follow-up rate: 77.7%) in different subgroups (2010–2018). *Bonferroni-adjusted (factor 3) for multiple testing.

**Figure 15:**
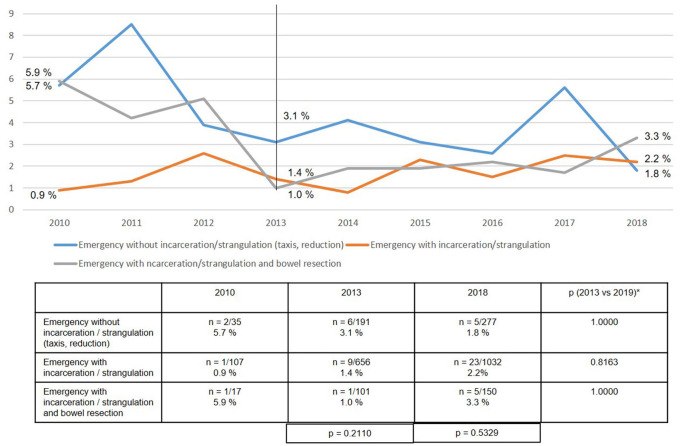
Chronic pain requiring treatment at 1-year follow-up of emergency inguinal hernia patients (*n* = 8,303 follow-up rate: 77.7%) in different subgroups (2010–2018). *Bonferroni-adjusted (factor 3) for multiple testing.

Surprisingly, no highly significant differences were found in either the recurrence rates or the rates of chronic pain requiring treatment at 1-year follow-up between years 2013 and 2018 (Figures 14, 15).

## Discussion

The present analysis of data from the Herniamed Registry for the years 2010 to 2019 identified a proportion of 2.7% of emergency admissions with operation within 24 h. The corresponding rate given in the literature for developed western countries is 2.5–7.7% (6).

The proportion of women with emergency groin hernia repairs remains unchanged at above 30%. The proportion of women with elective groin hernia repair is reported to be 8.0–11.5% (17, 19). The percentage of femoral hernias identified in the registry analysis was around 16%. While the risk of emergency groin hernia repair in women with femoral hernia is as high as 40.6% (19), this alone does not explain the high proportion of women with emergency groin hernia repairs. Hence, other influence factors are thought to contribute to the high rate of emergency groin hernia repairs in women. In the literature high age, obesity, higher ASA score, and recurrence, in addition to femoral hernia, are reported to lead to a higher rate of emergency groin hernia repairs (20).

Compared with elective groin hernia repair at 11–25% (17, 21), for emergency repair at 31% the registry analysis identified a higher number of larger defects as classified by European Hernia Society (EHS III: >3 cm) (22). Likewise, the proportion of scrotal hernias at 5.5% was higher for the emergency groin hernia repairs than the elective procedures at 2.7% (23). Hence, in the presence of the risk factors outlined here surgery should be indicated and watchful waiting practiced only with extreme caution, as otherwise the risk of needing emergency operations would rise (24).

In a Consensus Development Conference of the European Association of Endoscopic Surgery (EAES) and in the Guidelines of the International Endohernia Society a laparo-endoscopic procedure is recommended for treatment of incarcerated or strangulated groin hernia (25, 26). The advantage conferred by the laparo-endoscopic technique is that it allows exploration and reduction of the hernia sac contents, after which the bowel can be inspected and the blood flow assessed (25, 26). In a systematic review of the use of the laparo-endoscopic technique in emergency groin hernia repair, the rate of bowel resections was reported to be 5.2% (*n* = 17/328) (27). Based on the guideline recommendations and the findings of that systematic review, the laparo-endoscopic technique with abdominal exploration was adopted into the concept of a tailored approach (28). Following reduction of the hernia sac contents and possibly bowel resection, the degree of contamination will determine the groin hernia repair technique to be chosen (25–28).

Over the entire period from 2010 to 2019 the open Lichtenstein technique was used in 40.1% of emergency groin hernia repairs, followed by TAPP at 29.7% and TEP at 9.2%. The open Shouldice technique was only used in 3.8% of cases. Hence, the vast majority of techniques used were open mesh and laparo-endoscopic techniques. At least for emergency groin hernia repairs without bowel resection, a systematic review demonstrated the advantages conferred by the use of a mesh on the recurrence rate, without too high a risk of post-operative complications (29).

The registry analysis demonstrated that between 2013 and 2019 the use of the TAPP technique increased significantly from 21.9 to 38.0% in accordance with the guideline recommendations. The proportion of TEP techniques remained constant at around 10%, while that of the Lichtenstein technique declined somewhat. As such, a mesh was used in more than 85 % of emergency groin hernia repairs.

If one breaks down the total collective of emergency groin hernia repairs into the three subgroups, i.e., after reduction/ taxis (21%), without bowel resection (70%), and with bowel resection (9%), one notes that there are considerable differences in the surgical techniques used. Whereas, the proportion of laparo-endoscopic surgical techniques used in patients following reduction (taxis) of the hernia sac contents was over 60%, in the subgroup with incarcerated or strangulated groin hernia without bowel resection it was around 47%, and in the subgroup with bowel resection around 25%. In settings where bowel resection was needed, around 50% of the surgical techniques used are not recommended in the guidelines (15).

The perioperative outcomes of emergency groin hernia repair differed highly significantly between the subgroups. Patients, who despite emergency admission to hospital and operation within 24 h experienced successful reduction (taxis) of the hernia sac contents, had the lowest perioperative complication rates. Bearing in mind the patient's overall situation, reduction (taxis) of the hernia sac contents should therefore be attempted whenever possible. This can also be done under anesthesia (4, 5). Successful reduction of the incarcerated or strangulated hernia sac contents markedly improves the patient's prognosis.

If reduction (taxis) of the hernia sac contents is not successful, the need for bowel resection will determine the perioperative prognosis. Bowel resection results in a considerably poorer perioperative outcome with a mortality rate of 7.5%. In the literature the mortality rate for elective groin hernia repair is reported to be 0.1% (2). This once again underscores the importance of explorative laparoscopy with the possibility of reduction of the hernia sac contents and safe assessment of the blood flow to the strangulated bowel segment. Preservation of the strangulated bowel is the most important aspect when treating an incarcerated or strangulated groin hernia. The need for bowel resection carries a dramatically poorer prognosis for the patient, with less focus now on treatment of the hernia.

In addition to the subgroups of emergency groin hernia repair, perioperative complications are highly significantly negatively influenced by female gender and age ≥66 years (30).

If the patient survives emergency groin hernia repair and the hernia has been appropriately treated, the 1-year follow-up outcomes are surprising good in terms of the recurrence rate and the rate of chronic pain requiring treatment. No highly significant differences are seen here between the subgroups. Longer follow-up results must, of course, be awaited here.

One limitation of any registry analysis is incorrect and missing data. A contract was made with every participating hospital or surgeon where the latter committed to ensuring complete and correct data entry. Patients who because of their overall situation could not be properly informed about inclusion in the registry or who were not capable of giving their written consent were not included. At the time of certification of hernia centers the auditor must be informed about those cases that could not be documented in the registry. Comparison of the registry data with the literature data is an important aspect. Any deviations must be investigated once again for data soundness. Our data are in accordance with the literature. Another limitation of registry analysis is loss of follow-up information for around more than 20% of patients.

In summary, it can be stated that the patient collective with emergency admission and operation within 24 h for incarcerated or strangulated groin hernia should be analyzed as separate subgroups because of the considerable differences in prognosis between the subgroups with reduction (taxis) of the hernia sac contents, existing incarceration or strangulation without the need for bowel resection and patients with bowel resection. Defects >3 cm as well as femoral and scrotal defect localizations are at higher risk of emergency groin hernia repair. Women, too, are placed at an additionally higher risk of emergency because of femoral hernia.

Explorative laparoscopy and reduction of the hernia sac contents should always be undertaken to avoid bowel resection with its poor prognosis. Laparoscopy permits optimal assessment of bowel perfusion. The need for bowel resection carries a dramatically poorer perioperative prognosis with a mortality rate of 7.5%. Other factors influencing poor outcome are female gender and age ≤ 66 years. If the patient survives the acute situation, the 1-year follow-up outcomes are relatively good with regard to the recurrence rate and the rate of chronic pain requiring treatment.

## Data Availability Statement

The original contributions presented in the study are included in the article/supplementary material, further inquiries can be directed to the corresponding author/s.

## Ethics Statement

For this retrospective analysis of routine hernia surgery data from the Herniamed Registry ethical approval was not required. Written informed consent to participate in the Herniamed Registry was provided by all participants.

## Author Contributions

FK study concept, literature review, manuscript writing, revision of manuscript, and final approval of the manuscript. TH literature search and preparation of data for statistical analysis. DA statistical analysis, manuscript revision, and final approval of the manuscript. KZ, DW, BL, FM, WR, and DJ study concept, revision of manuscript, final approval of the manuscript. All authors contributed to the article and approved the submitted version.

## Conflict of Interest

FK reports grants to fund Herniamed from Johnson & Johnson, Norderstedt, grants from Karl Storz, Tuttlingen, grants from pfm medical, Cologne, grants from Dahlhausen, Cologne, grants from B Braun, Tuttlingen, grants from MenkeMed, Munich, grants from Bard, Karlsruhe, during the conduct of the study; personal fees from Bard, Karlsruhe, outside the submitted work. DA was employed by the company StatConsult GmbH. The remaining authors declare that the research was conducted in the absence of any commercial or financial relationships that could be construed as a potential conflict of interest.
